# Aquaporin 7 involved in GINSENOSIDE-RB1-mediated anti-obesity via peroxisome proliferator-activated receptor gamma pathway

**DOI:** 10.1186/s12986-020-00490-8

**Published:** 2020-08-17

**Authors:** Rong Guo, Lei Wang, Xianqin Zeng, Minghao Liu, Peng Zhou, Huixia Lu, Huili Lin, Mei Dong

**Affiliations:** 1 The Key Laboratory of Cardiovascular Remodeling and Function Research, Chinese Ministry of Education, Chinese National Health Commission and Chinese Academy of Medical Sciences, The State and Shandong Province Joint Key Laboratory of Translational Cardiovascular Medicine, Department of Cardiology, Qilu Hospital, Cheeloo College of Medicine, Shandong University, 107 Wenhuaxi Road, Jinan, 250012 China; 2 Department of Cardiology, The Second Affiliated Hospital of Fujian Medical University, Quanzhou, 362000 Fujian People’s Republic of China; 3 Department of Cardiology, Ji’an Municipal Center People’s Hospital, Ji’an, Jiangxi China; 4 State Key Laboratory of Cardiovascular Disease, Fuwai Hospital, National Center for Cardiovascular Diseases, Chinese Academy of Medical Sciences and Peking Union Medical College, Beijing, 100037 People’s Republic of China

**Keywords:** Ginsenoside Rb1, Obesity, Fat, Aquaporin 7, PPARγ, Lipid release

## Abstract

**Background:**

Obesity, characterized by the excessive accumulation of triglycerides in adipocytes and their decreased excretion from adipocytes, is closely related to various health problems. Ginsenoside Rb1 (Rb1), the most active component of the traditional Chinese medicine ginseng, has been reported to have positive effects on lipid metabolism. The aim of the present study was to determine the protective effects of Rb1 on glycolipid metabolism under obesity conditions and its mechanisms and to reveal the signaling pathways involved.

**Methods:**

In our study, male C57BL/6 mice with obesity induced by a high-fat diet (HFD) and mature 3 T3-L1 adipocytes were used to investigate the role of Rb1 in lipid accumulation and explore its possible molecular mechanism in vivo and in vitro, respectively.

**Results:**

Rb1 reduced the body weight, fat mass, adipocytes size and serum free fatty acid (FFA) concentration of obese mice. In differentiated 3 T3-L1 adipocytes, Rb1 reduced the accumulation of lipid droplets and stimulated output of triglycerides. Additionally, the expression of peroxisome proliferator-activated receptor gamma (PPARγ), phosphorylated PPARγ (Ser112) and aquaporin 7 (AQP7) was upregulated in adipocytes and adipose tissues upon Rb1 treatment. However, intervention of GW9662, PPARγ antagonist, attenuated Rb1-mediated effects on glycolipid metabolism and AQP7 levels.

**Conclusions:**

These data indicated that Rb1 reduced body weight and improved glycolipid metabolism by upregulating PPARγ and AQP7 protein levels. Our study indicated a potential role for Rb1 in the prevention and treatment of obesity.

## Background

Obesity, an increasing global public health issue, serves as a risk factor for an expanding set of metabolic diseases, including cardiovascular disease and type 2 diabetes [1]. The pathophysiological progression of obesity is related to increased lipids, mainly triglycerides, the hyperplastic and hypertrophic growth of adipocytes, and the expansion of adipose tissues especially white adipose tissue [2, 3]. Adipose tissue mass is determined by the storage of triglycerides in adipocytes and their removal from adipocytes. High storage but low removal of triglyceride promotes fat tissue accumulation and obesity [4]. This demonstrates that promoting the rate of triglyceride removal from adipocytes is an important antiobesity therapeutic target.

Aquaporins (AQPs) are transmembrane proteins that facilitate the permeation of water and small solutes across membranes, driven by osmotic of solute gradients [5]. Numerous studies have described the crucial role of aquaglyceroporins, a subfamily of AQPs, in adipose tissue biology and obesity onset [6, 7]. Under conditions of energy expenditure, triacylglycerol stored in adipocytes is hydrolyzed to glycerol and free fatty acids, which are released into the bloodstream [7]. Among the various AQPs, AQP7 is a vital glycerol transporter in adipocytes [8]. AQP7-depleted mice, showed progressive adipocyte hypertrophy, increased fat mass and metabolic disorders [9]. These effects are thought to be relevant to reduced adipocyte glycerol permeability and the subsequent accumulation of intracellular glycerol and triacylglycerol [10]. The modulation of adipocyte AQP7 expression has been proposed as a target in obesity therapy [11]. The upregulation of AQP7 expression or its functional activation might be an ideal approach for the treatment of obesity. AQP7 is a novel adipose-specific target gene of PPARγ [12]. AQP7 expression in adipocytes was reported to be increased by thiazolidinediones, which are synthetic PPARγ ligands. PPARγ regulates AQP7 expression through binding of the PPARγ-retinoid X receptor complex to the peroxisome proliferator-activated receptor response element (PPRE) region in the AQP7 gene promoter.

*Panax ginseng*, a traditional herbal medicine, has been widely used to treat various diseases in Eastern Asia for more than 4000 years. Ginsenoside Rb1 is the most abundant active component of *Panax ginseng*, has been reported to decrease body weight, ameliorate glycolipid metabolism and reduce triglyceride accumulation [13–15]. In addition, PPARγ was reported to be involved in the effects of Rb1 on adipocytes and adipose tissue. Although these studies implied that Rb1 can reduce fat mass and body weight, its molecular mechanism still remains unclear. Our previous studies also have revealed that Rb1 reduced lipid accumulation in macrophage foam cells [16], and improved the metabolic environment by inhibiting inflammatory reactions in atherosclerosis models [17].

However, whether Rb1 can regulate AQP7 expression through PPARγ to promote lipid transport and reduce body weight remains unclear.

## Methods

### Animal care and treatment

A total of 90 C57BL/6 mice (male, 4–5 weeks old) were obtained from Charles River Laboratories Animal Technology Company (Beijing, China). All mice were raised on a 12-h light/12-h dark cycle. After one week of adaptation, the mice were randomly divided into two groups and received different diets. Some mice were fed a normal diet (20.6% kcal from protein, 67.4% kcal from carbohydrate and 12% kcal from fat)(*n* = 30), and the rest were fed a high-fat diet (HFD, 20% kcal from protein, 20% kcal from carbohydrate and 60% kcal from fat (soybean oil and lard)) (*n* = 60) for 12 weeks. Mice fed a normal diet were randomly divided into 2 subgroups (*n* = 15 per group): the Chow group (fed a normal diet and treated with saline) and Chow+Rb1 group (fed a normal diet and treated with Rb1). The body weights were measured weekly. Mice fed a HFD were confirmed as obese if their weight was 20% greater than that of mice in the Chow group. Subsequently, mice fed a HFD were randomly divided into 4 subgroups (*n* = 15 per group): the HFD group, HFD + Rb1 group, HFD + GW9662 + Rb1 group and HFD + dimethylsulfoxide (DMSO) + Rb1 group. All groups treated with Rb1 were administered 40 mg/kg/d Rb1 (B21050, Shyuanye, China). Mice in the HFD group received an equal volume of saline. DMSO (D8371, Solarbio Beijing, China) was diluted to 1% in normal saline, and GW9662 (M6191, Sigma US) was prepared in 1% DMSO before administration. All treatments were conducted by daily intraperitoneal injection for 5 weeks. The injection volume of each liquid was 300 μl. GW9662 and DMSO treatments were administered 0.5 h later than Rb1 treatment, and mice in the HFD + Rb1 group were administered 300 μl of normal saline after injection of 300 μl of Rb1. After 5 weeks of treatment, the mice were euthanized. Subcutaneous, visceral and epididymal adipose tissues were harvested, and the weight of the fat mass divided by the body weight of mice was considered the body fat rate (BFR).

### Glucose tolerance test

The intraperitoneal glucose tolerance test (IPGTT) was conducted on the 16th week. After fasting overnight, mice were injected intraperitoneally with glucose at a dose of 2 g/kg body weight. Blood glucose concentrations in blood from the tail vein of the mice were measured at 0(fasting), 15, 30, 60 and 120 min after glucose administration.

### Serum biochemical analysis

Serum collected from each mouse was centrifuged at 3000 rpm for 15 min and stored at − 80 °C for further analysis. Blood biochemical tests to detect serum lipids (cholesterol, triglyceride, high density lipoprotein cholesterol, low density lipoprotein cholesterol, and free fatty acid) and blood glucose were performed as described in our previous report [18].

### Histological analysis

Adipose tissue was fixed overnight in 4% paraformaldehyde, and then embedded in paraffin. Tissue sections (5 μm) were collected and mounted onto slides. The sections were stained with hematoxylin and eosin and photographed at 100× magnification. Using Image-Pro Plus 6.0 software (IPP 6.0, Media Cybernetics, Rockville, MD, United States), at least two fields per slice and six slices per fat mass were analyzed to quantify adipocyte size.

### Cell culture and treatment

Mouse 3 T3-L1 preadipocytes were purchased from American Type Culture Collection (ATCC, US). The cells were cultured in high glucose Dulbecco’s modified Eagle medium (DMEM) with 10% fetal bovine serum (FBS) at 37 °C in a saturated humidity atmosphere of 5% CO_2_. The classic cocktail method was used to induce 3 T3-L1 preadipocyte differentiation. Briefly, two days after reaching contact inhibition, cells were treated with a mixture of 0.5 mM 3-isobutyl-1-methylxanthine, 1 μM dexamethasone, and 10 μg/mL insulin in DMEM containing 10% FBS for 2 days. The media were replaced with DMEM containing 10 μg/mL insulin and 10% FBS, after which cells were incubated for another 2 days, and the growth media used for an additional day consisted of only DMEM with 10% FBS. When more than 90% of the 3 T3-L1 preadipocytes had been induced into mature 3 T3-L1 adipocytes with accumulated lipid droplets in the cytoplasm, they could be used for further study. The optimum dose of Rb1 was 40 μM, which was determined by titration with different concentrations of Rb1 for 24 h. The dose of GW9662 used was 5 mM, and the dose of DMSO used was 0.01%.

### Oil red O staining and triglyceride assay

Lipid droplets in the cytoplasm were measured using oil red O staining. Differentiated 3 T3-L1 adipocytes were placed on cover slides in plates and treated with the corresponding drug for 24 h. Cells were washed with phosphate-buffered saline two times and fixed with 4% paraformaldehyde for 2 h at room temperature. The culture supernatant was collected and used to detect the content of free triglycerides, leptin and adiponectin with an ELISA kit (E1003, Applygen, China). Fixed cells were washed once with distilled water and 60% isopropanol. Subsequently, 0.5% oil red O was added to the cells and incubated for 1 h. The surface dye was rinsed away with distilled water. Cells were observed under a microscope at 100× magnification.

### Western blot analysis

The total protein was extracted from 3 T3-L1 adipocytes and mouse epididymal adipose tissues with a total protein extraction kit (P1250, Applygen). Protein expression levels of AQP7 and PPARγ were detected. Samples were separated by 10% SDS-PAGE and transferred onto 0.45 μm PVDF membranes (Bio-Rad). The membranes were blocked in 5% nonfat dry milk followed by incubation with primary antibodies against AQP7 (AB15568, Merck-Millipore, Germany), PPARγ (ab45036, Abcam, UK), phosphorylated peroxisome proliferator-activated receptor γ (pPPARγ, Ser112) (abs130911a, Absin, China) and Tubulin-α (AF7010, Affinity, US) overnight at 4 °C. The secondary antibody (ZSGB-BIO, China) was goat anti-rabbit immunoglobulin G antibody. After washing, the immunocomplex was incubated with secondary antibody (1:10,000) for 2 h. Bands were visualized by chemiluminescence (Millipore Corporation, Billerica, MA, USA).

### Statistical analysis

All results are expressed as the means±SEMs. Multiple group comparisons were analyzed by ANOVA followed by Tukey’s post hoc test, and the significance of differences between the mean values of two groups were analyzed using the unpaired two-tailed Student’s *t*-test. Statistical significance was defined as *p* < 0.05.

## Results

### Rb1 reduced body weight and fat mass partially in a PPARγ-dependent manner

**Fig. 1:**
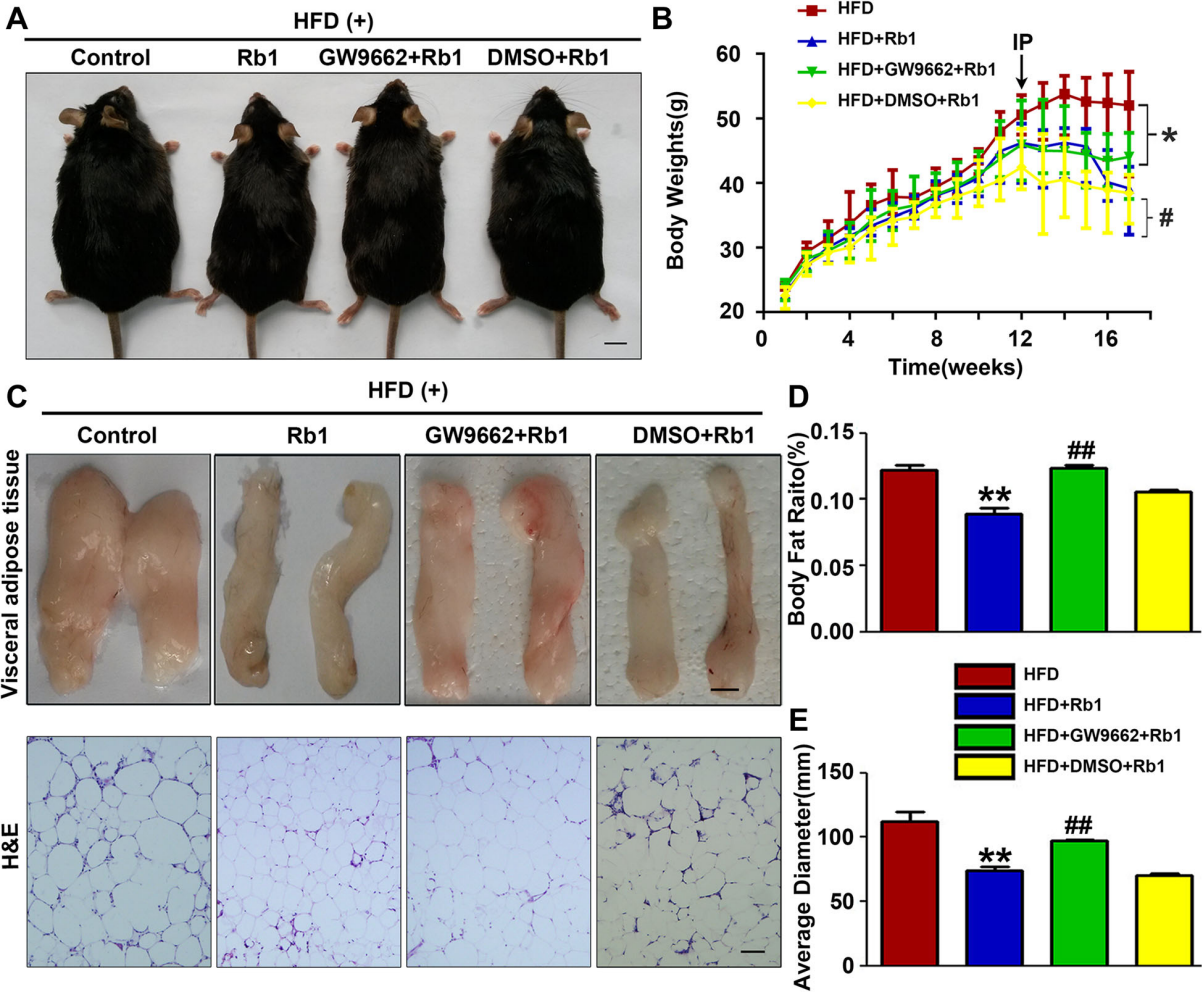
Effects of Rb1 and/or GW9662 on body weight and fat mass in HFD-induced obese mice. **a** Representative individuals from the different groups. Scale bar: 1 cm. **b** Variations in the body weight of the four groups over weeks. **c** En face view of visceral adipose tissue from representative individuals from the four groups. Scale bar: 5mm. H&E staining of visceral fat tissues. Scale bar: 50 μm. H&E staining of visceral fat tissues. Scale bar: 50 μm. **d** Quantification of the body fat rate. **e** Quantification of the average adipocyte diameter. IP: intraperitoneal injection. *p < 0.05, the HFD + Rb1 group compared with the HFD group; ^#^*p* < 0.05, the HFD + GW9662 + Rb1 group compared with the HFD + Rb1 group; **p < 0.01, the HFD + Rb1 group compared with the HFD group; ^##^*p* < 0.01, the HFD + GW9662 + Rb1 group compared with the HFD + Rb1 group

To confirm the effects of Rb1 on body weight in obese mice, we established a mouse model of obesity through HFD feeding. After 12 weeks of HFD feeding, the body weights of the mice were significantly increased compared with those of mice in the Chow group (*p* < 0.01) (Supplement 1) and reached the standard for obesity [19]. The weights of all HFD-fed mice were measured weekly to observe trends over 17 weeks. Then, we investigated the molecular mechanisms of this change. Studies revealed that PPARγ in adipocytes could be activated by Rb1 treatment [20, 21]. To further determine the role of PPARγ in Rb1-induced body weight and fat mass loss, mice were intraperitoneally injected with GW9662, an antagonist of PPARγ. The results showed that Rb1 treatment markedly reduced the body weights of obese mice (Fig. 1a-b) and decreased fat mass accumulation (Fig. 1c). BFR (body fat rate = weight of fat mass/body weight*100%) was significantly reduced in the Rb1-treated group (Fig. 1d). H&E staining showed that the in vivo adipocyte size in the HFD + Rb1 group mice was markedly reduced compared with that in the control group (Fig. 1c-e). However, GW9662 treatment partially reversed the decrease in body weight, fat mass and adipocyte size induced by Rb1. There was no significant difference in these parameters in the presence and absence of DMSO, the PPARγ dissolvent. These data suggested that the PPARγ pathway is involved in the effects of Rb1 in reducing body weight and fat mass in HFD-induced obese mice.

### Rb1 improved the glucose tolerance and influenced the serum lipid profile of obese mice

**Fig. 2:**
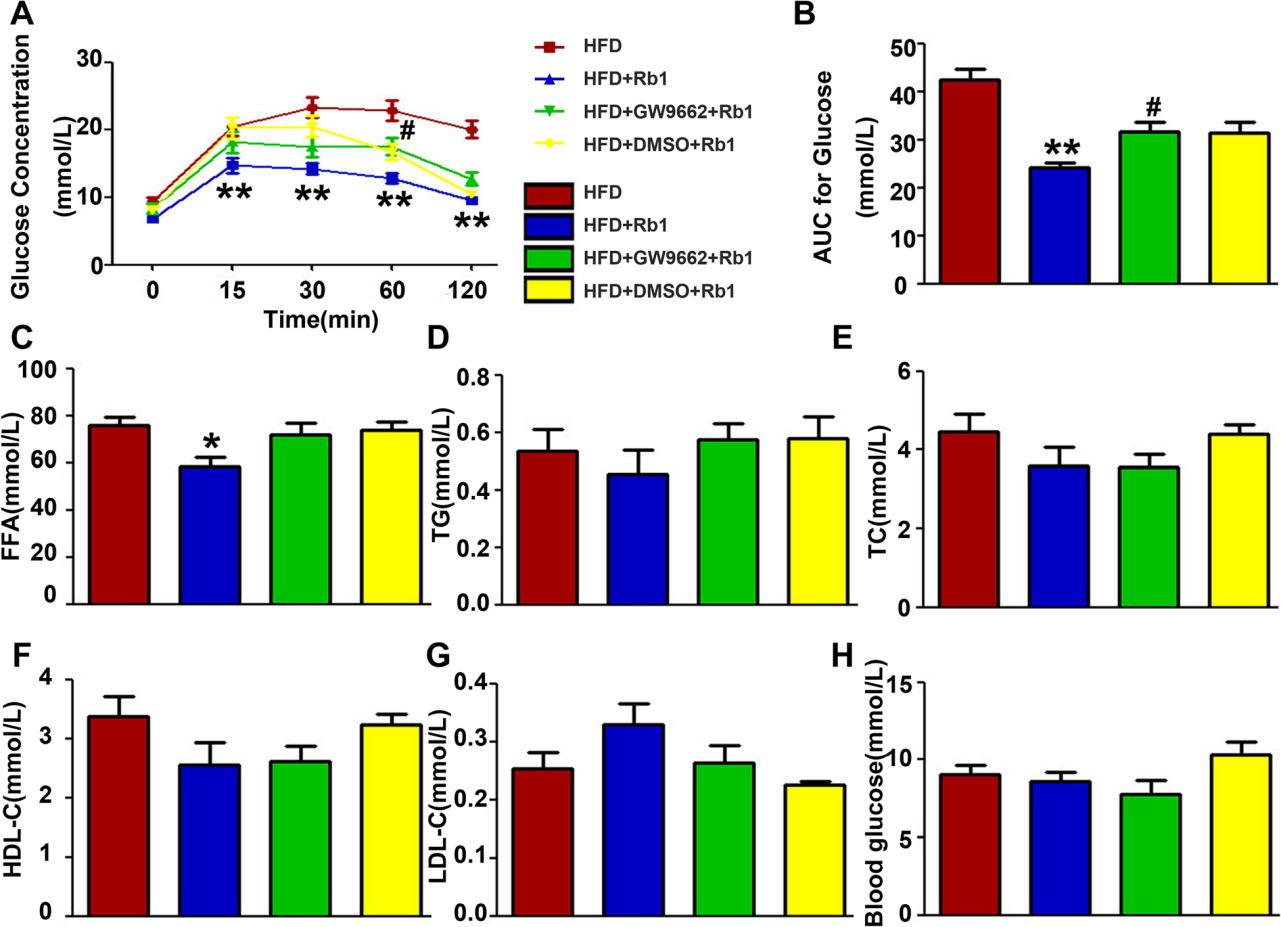
Effects of Rb1 on glycolipid metabolism. **a** Blood glucose detected by the IPGTT from the tail vein at different time points after glucose injection. **b** Quantification of the AUC of the IPGTT of mice of different groups. **c** The serum levels of FFAs, **d** TGs, **e** TC, **f** HDL-C, **g** LDL-C and **h** glucose in obese mice under different treatment conditions. *p < 0.05, the HFD + Rb1 group compared with the HFD group; **p < 0.01, the HFD + Rb1 group compared with the HFD group; ^#^*p* < 0.05, the HFD + GW9662 + Rb1 group compared with the HFD + Rb1 group

As glycolipid metabolism plays a vital role in body weight growth, we investigated whether Rb1 affects glycolipid metabolism. Serum concertrations of cholesterol (TC), triglycerides (TGs), high-density lipoprotein cholesterol (HDL-C), low-density lipoprotein cholesterol (LDL-C), FFAs and blood glucose were detected in HFD-fed mice. The IPGTT was carried out on the 16th week. The area under the curve (AUC) of the IPGTT in the Rb1 group was reduced compared to that in the HFD group. The AUC of the IPGTT was significantly higher in the GW9662 + Rb1 group than in the Rb1 group, and there was no difference in the AUC of the IPGTT between the HFD + DMSO+Rb1 and HFD + Rb1 groups (Fig. 2a-b). Our results showed that Rb1 contributed to the reduced serum FFA levels (Fig. 2c). Serum TG levels tended to decrease with Rb1 treatment, although this difference did not achieve statistical significance. This effect was abrogated by GW9662(Fig. 2d). Serum TC, HDL-C, LDL-C and blood glucose levels were not affected by Rb1 treatment (Fig. 2e-h). In view of these results, Rb1 efficiently lowered the AUC of the IPGTT and improved the ability of obese mice to regulate blood glucose and TG. Inhibition of PPARγ with its antagonist partly reversed this impact. Rb1 decreased the serum level of FFAs, but there was no evidence that PPARγ was involved in the mechanism.

### Rb1 augmented AQP7 expression in adipose tissue via the PPARγ Signaling pathway

**Fig. 3:**
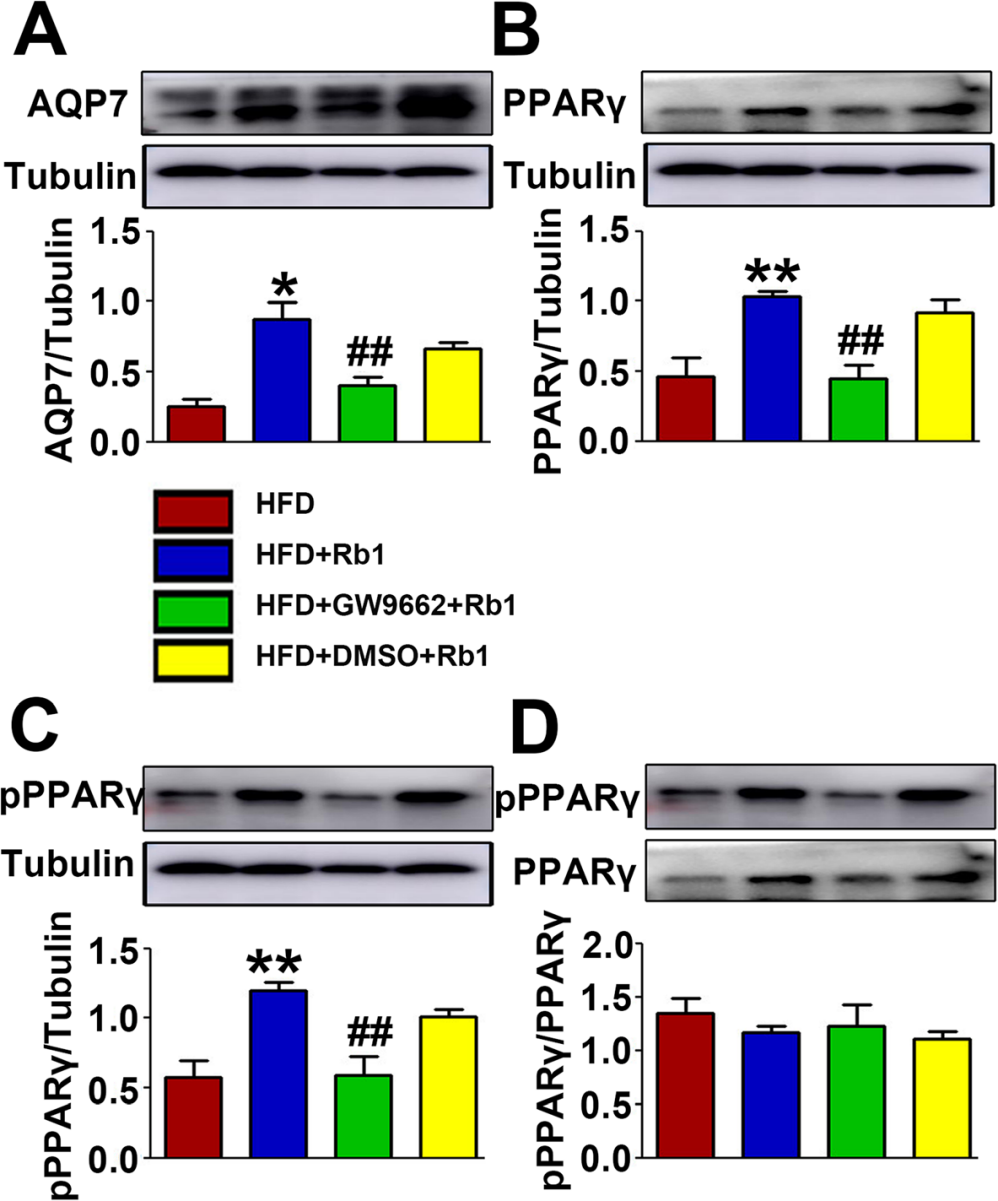
Rb1 increased the expression levels of AQP7 and PPARγ in adipose tissue. **a** Representative western blot analysis and quantification of AQP7. **b** Representative western blot analysis and quantification of PPARγ. **c** Representative western blot analysis and quantification of pPPARγ. **d** Representative western blot analysis and quantification of pPPARγ/PPARγ in vivo. *p < 0.05, the HFD + Rb1 group compared with the HFD group; **p < 0.01, the HFD + Rb1 group compared with the HFD group; ^##^*p* < 0.01, the HFD + GW9662 + Rb1 group compared with the HFD + Rb1 group

AQP7 is a membrane channel that tranports glycerol and interferes with triglyceride accumulation in adipocytes [22]. It is a target gene of PPARγ and can be activated by PPARγ agonists [12, 23]. To investigate the effect of Rb1 on AQP7 expression and evaluate the involvement of PPARγ, we collected visceral adipose tissue and extracted proteins from the tissue. Rb1 promoted the expression of AQP7, but this effect was partially reversed by a PPARγ inhibitor in vivo (Fig. 3a). Meanwhile, Rb1 also upregulated the expression of PPARγ (Fig. 3b) and pPPARγ (Fig. 3c), and these effects were inhibited by GW9662. The ratio between PPARγ and pPPARγ expression did not change significantly with different treatments (Fig. 3d). These results indicated that Rb1 increased AQP7 expression in a PPARγ-dependent manner, which was possibly responsible for its function in inhibiting adipose tissue expansion and reducing body weight. The improvement of glycolipid metabolism with Rb1 treatment might be associated with the upregulation of PPARγ and AQP7.

### Rb1 induced AQP7 and PPARγ expression in mature 3 T3-L1 adipocytes

**Fig. 4:**
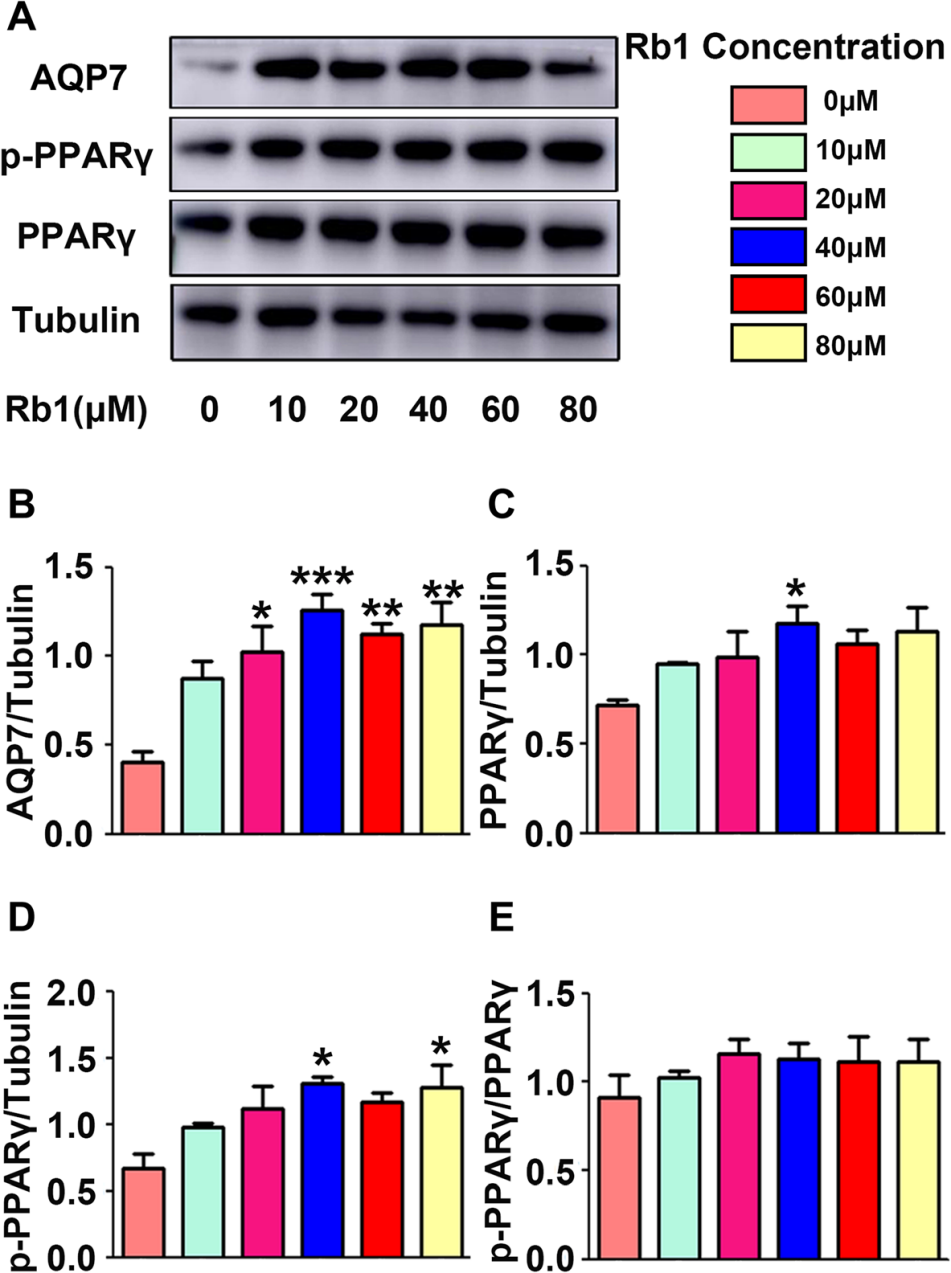
AQP7, pPPARγ and PPARγ expression in mature 3 T3-L1 adipocytes treated with Rb1 at different concentrations. **a** Representative western blot analysis of AQP7, pPPARγ and PPARγ expression in mature 3 T3-L1 adipocytes treated with Rb1 at different concentrations. **b** Quantification of AQP7, **c** PPARγ, **d** pPPARγ and **e** pPPARγ/PPARγ expression relative to the tubulin level in different groups. *p < 0.05, compared with the control group (0 μM Rb1); **p < 0.01, compared with the control group (0 μM Rb1), ***p < 0.001, compared with the control group (0 μM Rb1)

To explore the role of Rb1 in lipid metabolism in vitro, the expression of AQP7, PPARγ and pPPARγ was detected in mature 3 T3-L1 adipocytes with or without Rb1 treatment. Differentiated 3 T3-L1 adipocytes were treated with Rb1 at different concentrations for 24 h. Our results showed that AQP7 protein levels were upregulated under treatment with 20 to 80 μM Rb1 (Fig. 4a-b). The PPARγ protein level was increased with 40 μM Rb1 treatment (Fig. 4c) and pPPARγ level was increased with 40 μM and 80 μM Rb1 treatment (Fig. 4d) compared to that in the control group (0 μM Rb1). However, the pPPARγ/PPARγ level was unchanged with Rb1 treatment (Fig. 4e). We selected 40 μM Rb1 for use in the following experiments.

### Rb1 decreased lipid storage in adipocytes by promoting PPARγ/ AQP7 protein expression in mature 3 T3-L1 adipocytes

**Fig. 5:**
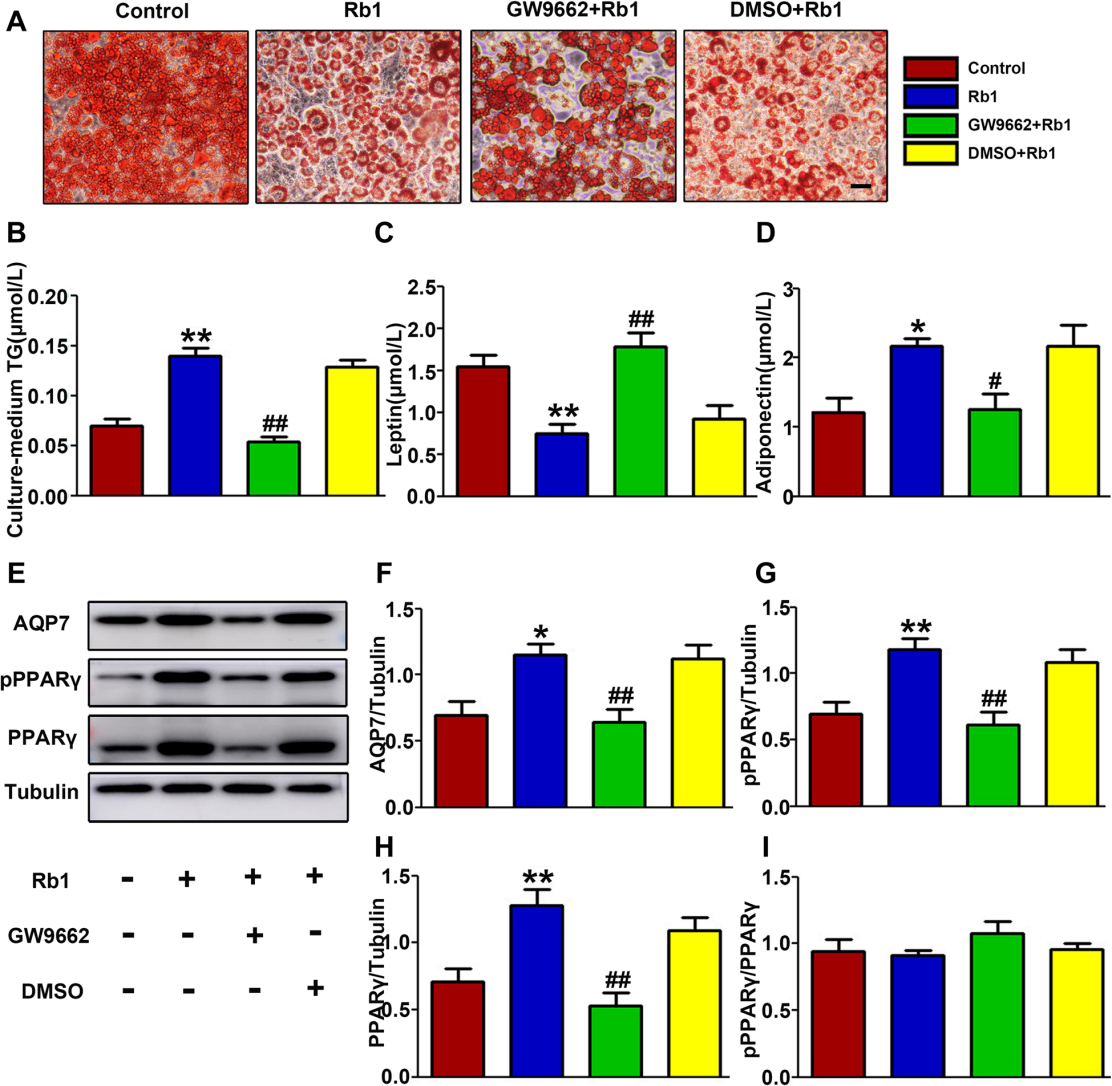
Rb1 decreased the lipid storage inside differentiated 3 T3-L1 adipocytes. **a** Representative oil red O staining of adipocytes with or without Rb1 treatment in the presence or absence of GW9662. Scale bar: 50 μm. **b** Quantification of the TG concentration, **c** leptin levels and **d** adiponectin expression levels in the cell culture medium. **e** Representative western blot analysis and **f** quantification of AQP7, **g** pPPARγ, **h** PPARγ and **i** pPPARγ/PPARγ in vitro. *p < 0.05, the Rb1 group compared with the control group; **p < 0.01, the Rb1 group compared with the control group; ^#^*p* < 0.05, the GW9662 + Rb1 group compared with the Rb1 group; ^##^*p* < 0.01, the GW9662 + Rb1 group compared with the Rb1 group

To further understand the molecular mechanism by which Rb1 regulates AQP7, we inhibited PPARγ by incubation with GW9662 in vitro. Oil red O staining showed that the intracellular lipid droplet size was significantly decreased in the Rb1 group (Fig. 5a). The triglyceride concentration in the cell medium was increased with Rb1 treatment (Fig. 5b). Compared to the Rb1 group, the GW9662 + Rb1 group showed larger adipocytes lipids and a lower triglyceride content in the medium. As an endocrine organ, adipose tissue secretes many adipocytokines [24]. Leptin and adiponectin, typical adipokines, play important roles in lipodystrophy. The expression of adiponectin decreases with an increase in adiposity, while leptin levels increase in obesity [25]. To obtain a deeper understanding of the effects of Rb1, we measured the concentrations of leptin and adiponectin in adipocytes. Leptin levels in the culture supernatant were decreased by Rb1 treatment (Fig. 5c). Rb1 increased adiponectin levels in the cell culture medium of adipocytes (Fig. 5d). These effects of Rb1 on leptin and adiponectin could be inhibited by GW9662. These results showed that Rb1 could influence the metabolic state of adipocytes through the PPARγ pathway.

Our results indicated that Rb1 augmented AQP7 protein expression in adipocytes, which was suppressed by a PPARγ antagonist (Fig. 5e-f). This result was consistent with the in-vivo results described above (Fig. 3a). The protein levels of both pPPARγ and PPARγ were increased in the Rb1 group (Fig. 5g-h). There was no significant difference in the pPPARγ/PPARγ ratio among the groups (Fig. 5i). Taken together, these results indicated that Rb1 attenuated lipid storage, promoted lipid excretion and influenced adipokine levels in adipocytes through the PPARγ-AQP7 pathway.

## Discussion

In this study, we found that Rb1 treatment reduced body weight, diminished fat mass and adipocyte enlargement and improved glycolipid metabolism in HFD-induced obese C57BL/6 mice. In differentiated 3 T3-L1 adipocytes, Rb1 decreased lipid droplet size. Rb1 augmented the expression levels of AQP7, influencing lipid metabolism both at both the animal and cellular levels. Rb1-mediated upregulation of the AQP7 protein was dependent on the PPARγ signaling pathway. A PPARγ inhibitor partially abrogated the beneficial effects of Rb1 on glycolipid metabolism. These results indicated that Rb1 promoted AQP7 expression via the PPARγ signaling pathway to ameliorate obesity status both in vivo and in vitro.

Obesity is closely associated with energy-balance dysregulation under the influence of environmental and genetic factors [2, 26]. The anti-obesity function of Rb1 might be related to energy balance. A study reported that administration of Rb1 significantly suppressed food intake and increased energy expenditure in HFD-induced obese mice [27]. Adipocyte hypertrophy and excessive adipose accumulation are biological characteristics of obesity. The principal metabolic comorbidity associated with excessive body fat is dyslipidemia [28]. Lipogenesis and lipolysis in adipocytes are essential processes for the maintenance of lipid homeostasis. Adipocytes, the main component of adipose tissues, continuously synthesize and hydrolyze triglycerides in response to energy balance.

Ginseng, the root of *Panax ginseng*, is believed to delay senility and promote longevity. Ginsenosides, the most biologically active components of ginseng, exert multiple beneficial effects in the circulatory, endocrine, immune and central nervous systems [29]. Rb1 is the most abundant and representative ginsenoside. We first demonstrated a new role for Rb1 against obesity. Our study shows that Rb1 significantly ameliorated glycolipid metabolism and diminished body weight without damaging liver function (Supplement 2). We have proven that Rb1 could significantly diminish the body weights of HFD-induced obese mice. According to previous studies, the possible molecular mechanisms of its anti-obesity effects may involve the phosphatidylinositol 3-kinase (PI3K)/Akt signaling pathway and neuropeptide Y in the central nervous system [13, 27].

Regulation of AQP7 in adipose tissue appears crucial for obesity treatment. Human and rodent adipose tissue expresses AQP7 mRNA, but not that of other AQPs, in high abundance. AQP7 is involved in the process of adipogenesis and the transport of triglycerides in adipocytes [30]. The downregulation of AQP7 expression is closely related to the occurrence of type 2 diabetes and obesity [31]. However, AQP7 expression varies in the different adipose depots of obese people [32]. A sex-related difference in the effects of exercise training on AQP7 were also observed, which might be correlated with estradiol [33]. Evidence regarding the relationship between AQP7 expression and type 2 diabetes is still lacking. In this study, we found that Rb1 increased AQP7 expression both in vitro and in vivo. Body weight and fat mass in obese mice and lipid accumulation in mature 3 T3-L1 adipocytes were decreased under Rb1 treatment. Increased AQP7 expression and improved glycometabolism were observed in obese mice. With the increase in AQP7, lipid storage inside adipocytes was significantly decreased by Rb1. In addition, serum TG levels tended to be decreased in obese mice with Rb1 treatment.

To date, the most common reported regulators of AQP7 are insulin, leptin, and PPARγ [12, 34]. With the suppression of AQP7, plasma leptin concentrations were shown to be positively correlated with body fat mass [35]. We revealed that Rb1 decreased the leptin concentration in cell culture medium of adipocytes, but whether this phenomenon is correlated with the change in AQP7 expression is still unknown. As a master gene of adipogenesis, PPARγ can upregulate the expression of AQP7. PPARγ, which is mainly expressed in adipose tissue and the immune system, is closely related to the differentiation of adipocytes, glycolipid metabolism and insulin resistance [36]. Thiazolidinediones, insulin sensitizers that stimulate PPARγ, increased AQP7 mRNA abundance in the adipose tissue of male rodents [37]. Our study demonstrated that Rb1 can regulate AQP7 and increase glycerol release from adipocytes by activating PPARγ. All effects of Rb1 on adipose tissue and adipocytes determined in this experiment could be partially reversed by GW9662, an inhibitor of PPARγ.

In addition to its genetic regulation, PPARγ undergoes a variety of posttranscriptional modifications. Both of these mechanisms determine its specific activities [38]. The posttranscriptional modifications of PPARγ include serine phosphorylation, acetylation and lysine sumoylation [39]. Phosphorylation of PPARγ at Ser273 is essential for insulin resistance related to obesity [40, 41]. Dephosphorylation of Ser112 is associated with adipogenesis, and the phosphorylation of PPAR at Ser112 strongly inhibits adipogenesis [42, 43]. Our study showed that Rb1 could promote the expression of pPPARγ (Ser112), but no significant increase in pPPARγ (Ser112) / PPARγ was observed. This finding suggested that Rb1 plays an important role in the upregulation of PPARγ rather than the phosphorylation of PPARγ.

Considering the pathophysiological feature of obesity, turnover of FFAs and glycerol is vital for glycolipid metabolism. Adipocyte glycerol permeability is a key element in the regulation of fat accumulation. An increase in FFAs can sensitively reflect dyslipidemia. Obese individuals produce excessive FFAs, which can accelerate the development of metabolic syndrome and cardiovascular disease. Wang et al. found that Rb1 could reduce oxidative stress and inflammation in 3 T3-L1 adipocytes by reducing the FFA content [44]. Shang et al. found that Rb1 could improve heterotopic lipid deposition and downregulate FFA levels in obese mice [45]. In this study, Rb1 treatment significantly reduced lipid accumulation and increased the secretion of TG in 3 T3-L1 adipocytes. In addition, Rb1 significantly reduced the plasma level of FFAs and the size of the adipocytes. These results showed that Rb1 has potential value in promoting the transport of lipids in adipocytes. However, there were no significant differences in the plasma levels of TC, HDL-C, LDL-C, TGs or blood sugar between the control group and the Rb1 group. This might be related to the complicated metabolic pathways in the lipids of different organs, as our experiment focused on only adipose tissue.

Intervention with Rb1 did not change serum lipid levels compared to those in the HFD group in this study, while triglyceride levels tended to be lower, but this difference was not significant. This finding seems inconsistent with previous studies [27]. Instead of ApoE-knockout mice, we used wild-type C57BL/6 mice, the lipids of which are hard to regulate. In addition, a 5-week intervention time maybe not long enough for Rb1 to significantly reverse lipid metabolism. In addition, drug delivery methods also influence intracellular concentrations in vivo.

## Conclusion

Our results indicate that ginsenoside Rb1 can promote lipid transport and ameliorate obesity by upregulating AQP7 through the PPARγ pathway. Our study suggests Rb1 as a promising antiobesity therapeutic through its targeting of the transportation of lipids.

## Supplementary information

**Additional file 1: Supplement 1**. Variations in the body weight of mice on different diets with or without Rb1 treatment. IP: intraperitoneal injection. **p < 0.01, the HFD group compared to the Chow group; ^#^*p* < 0.05, the HFD group compared to the HFD + Rb1 group.

**Additional file 2: Supplement 2**. Liver function in mice from different groups. (A) Quantification of ALT serum levels. (B) Quantification of AST serum levels. *p < 0.05, the HFD + Rb1 group compared to the HFD group; ^#^*p* < 0.05, the HFD + GW9662 + Rb1 compared to the HFD + Rb1 group.

## Data Availability

The datasets used or analyzed during the current study are available from the corresponding author on reasonable request.

## Abbreviations

Rb1: Ginsenoside Rb1
HFD: High-Fat Diet
FFA: Free Fatty Acid
PPARγ: Peroxisome Proliferator-activated Receptor Gamma
AQP7: Aquaporin 7
AQPs: Aquaporins
PPRE: Peroxisome Proliferator-activated Receptor Response Element
DMSO: Dimethylsulfoxide
BFR: Body Fat Rate
IPGTT: Intraperitoneal Glucose Tolerance Test
DMEM: Dulbecco’s Modified Eagle Medium
IBMX: 3-isobutyl-1-methylxanthine
pPPARγ: Phosphorylated Peroxisome Proliferators-Activated Receptor γ
TC: Cholesterol
TG: Triglyceride
HDL-C: High Density Lipoprotein Cholesterol
LDL-C: Low Density Lipoprotein Cholesterol
