# Supplementary figures and images for: Aquaporin 7 involved in GINSENOSIDE-RB1-mediated anti-obesity via peroxisome proliferator-activated receptor gamma pathway
Source: Nutr Metab (Lond). 2020 Aug 17;17:69. doi: 10.1186/s12986-020-00490-8 (PMC7433204; doi:10.1186/s12986-020-00490-8)

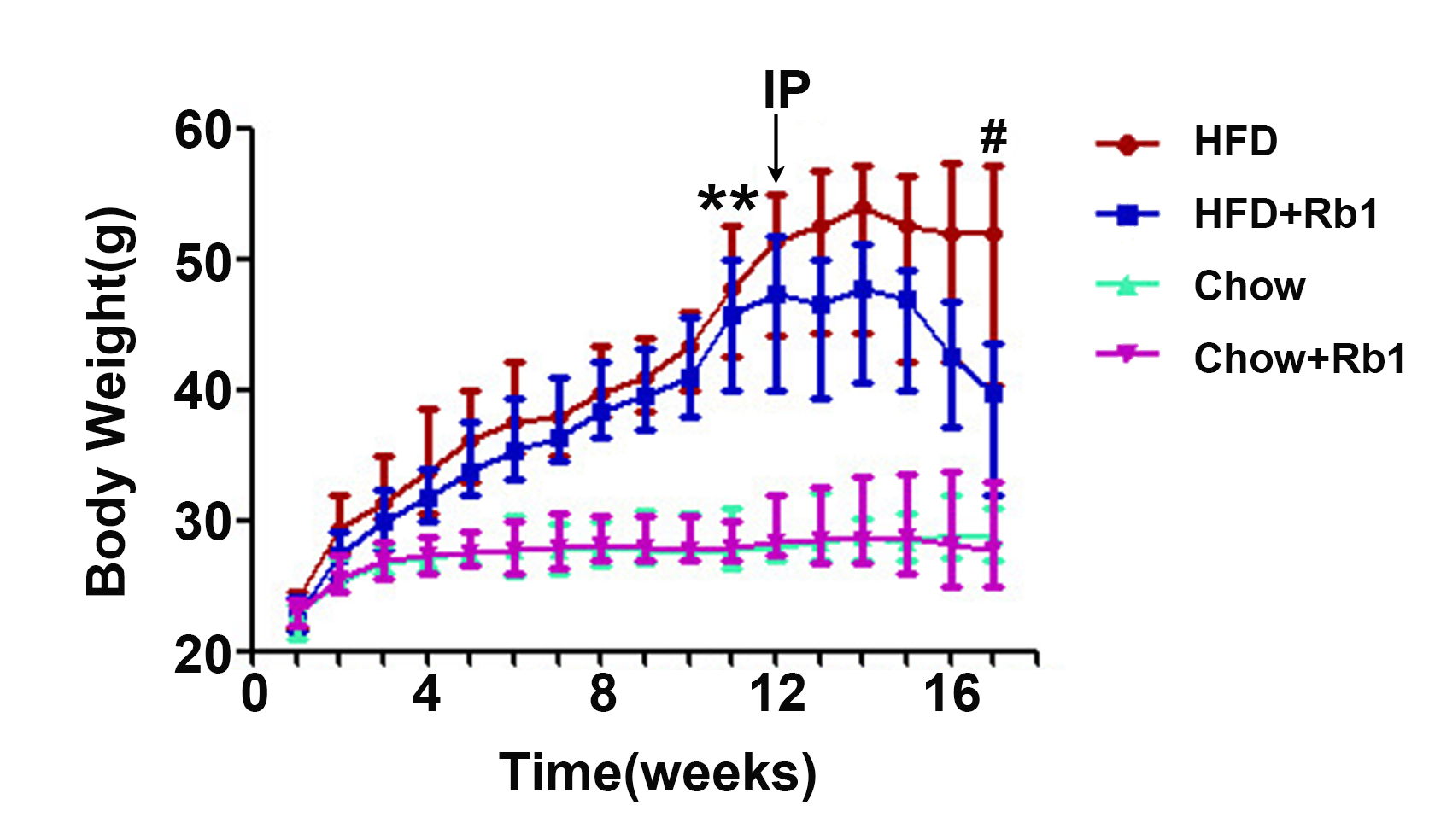

Supplement: Supplementary file 1 — Additional file 1: Supplement 1. Variations in the body weight of mice on different diets with or without Rb1 treatment. IP: intraperitoneal injection. **p < 0.01, the HFD group compared to the Chow group; #p < 0.05, the HFD group compared to the HFD + Rb1 group. [file 12986_2020_490_MOESM1_ESM.tif]

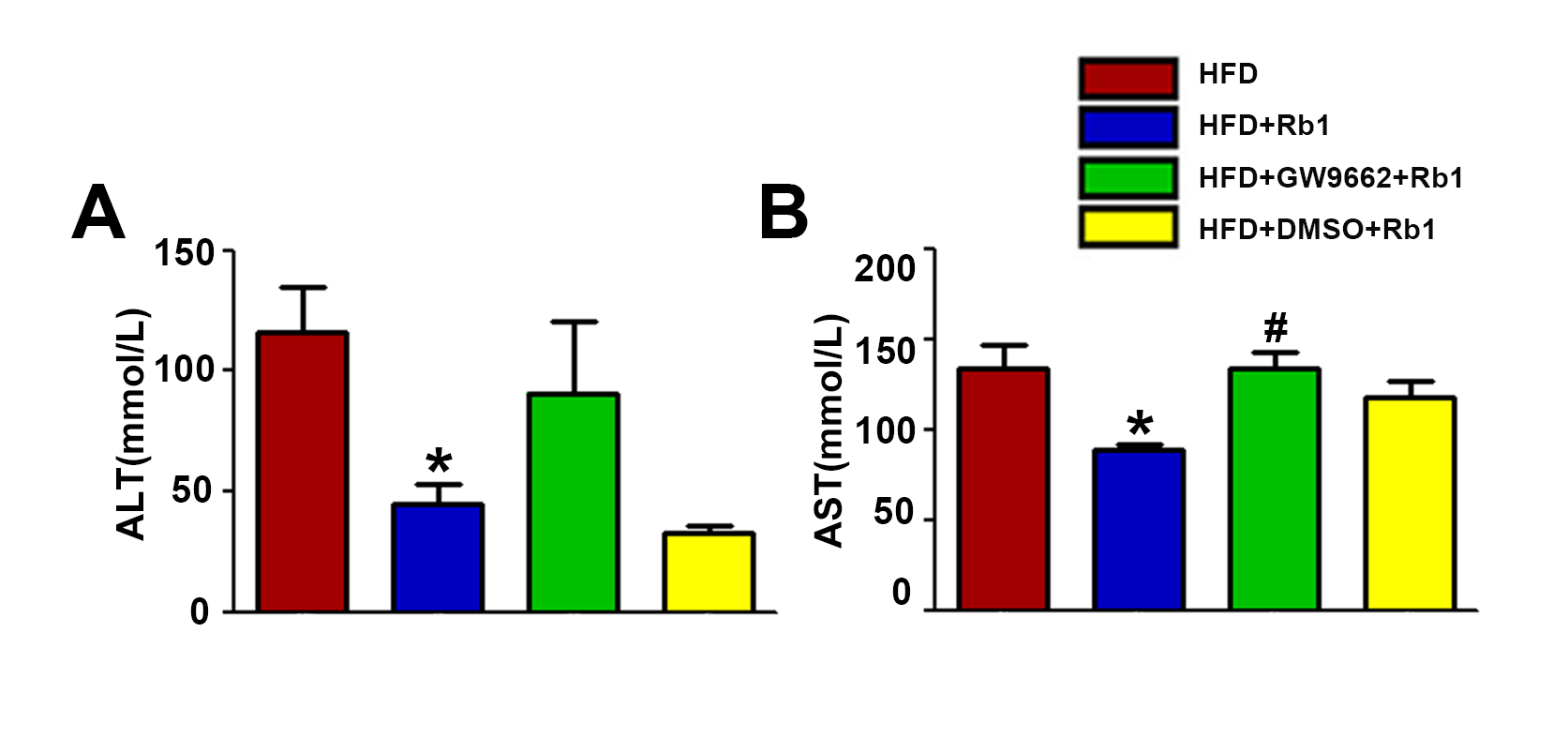

Supplement: Supplementary file 2 — Additional file 2: Supplement 2. Liver function in mice from different groups. (A) Quantification of ALT serum levels. (B) Quantification of AST serum levels. *p < 0.05, the HFD + Rb1 group compared to the HFD group; #p < 0.05, the HFD + GW9662 + Rb1 compared to the HFD + Rb1 group. [file 12986_2020_490_MOESM2_ESM.tif]
